# An Evaluation of the Wear Resistance of Electroplated Nickel Coatings Composited with 2,2,6,6-Tetramethylpiperidine 1-oxyl-Oxidized Cellulose Nanofibers

**DOI:** 10.3390/polym16020224

**Published:** 2024-01-12

**Authors:** Makoto Iioka, Wataru Kawanabe, Subaru Tsujimura, Tatsuya Kobayashi, Ikuo Shohji

**Affiliations:** 1 Graduate School of Science and Technology, Gunma University, Kiryu 376-8515, Japan; 2 School of Science and Technology, Gunma University, Kiryu 376-8515, Japan

**Keywords:** wear resistance, wear mechanism, composite coating, composite plating, cellulose nanofiber, TEMPO-oxidized cellulose nanofiber

## Abstract

In this study, the wear resistance of nickel (Ni)–cellulose nanofiber (CNF) composite electroplated films on steel plates (JIS SPCC, cold-rolled steel) was evaluated, including their surface and microstructural properties. In the CNF sample, 2,2,6,6-tetramethylpiperidine 1-oxyl (TEMPO)-oxidized CNF was used. As a result of the ball-on-disk abrasion test, in which steel (SUJ2) balls were used as the counterpart material, the plated film obtained with the addition of 1 g/L of CNF to the plating solution showed the highest wear resistance in this study. Compared to the conventional Ni-plated film without CNF, the abrasion loss volume on the plated side was reduced by approximately 79%, and that on the ball side was reduced remarkably by 94%. A microstructural analysis of the abrasion scars showed areas where co-deposited CNFs were stretched in the direction of abrasion, suggesting that the wear reduction effect was caused by sliding between the individual CNFs within the aggregates. Moreover, the hardness of the plated film increased when the Ni crystallite size became finer. It was confirmed that the co-deposition of fine CNFs is effective in improving hardness, whereas the co-deposition of a certain degree of aggregated CNFs is effective in exhibiting the wear reduction effect.

## 1. Introduction

We have been developing a new functional coating, a nickel (Ni)–cellulose nanofiber composite electroplated film, with sustainable properties [1,2,3,4]. Dispersion enhancement and self-lubricating properties are expected to be achieved by compounding cellulose nanofibers, which are renewable resources obtained from plants, are five times stronger than steel at one-fifth of the density, and are crystalline resin [5]. One of the application goals for this composite film is wear-resistant coatings.

Cellulose nanofibers can be used as a composite material, and, in particular, cellulose nanofiber composite resins have attracted a great deal of attention [6,7]. Cellulose nanofiber composite resins have shown better mechanical performance than the base polymer, making them suitable for various industries [3,8], such as the automotive [9], packaging [10], medical implant [11], electronics [12,13,14,15], building material [16], and paper industries [17]. However, very few studies have reported on composite materials with cellulose added to metal-based matrices. Recently, in 2022, Kurita et al. reported a titanium (Ti) sintered material with the addition of cellulose nanofibers [18]. The material was strengthened by the formation of titanium carbide from carbonized cellulose nanofibers during the sintering process, and the Ti–cellulose nanofiber composite material with 3.25 mass% cellulose nanofibers achieved a tensile strength of over 700 MPa, twice that of pure titanium [18]. In 2023, Osada et al. applied cellulose nanofibers as an additive to binder in metal powder injection molding of stainless steel, and they reported the effects of suppressing deformation during sintering and reducing the oxygen content of sintered material [19]. Our studies on Ni–cellulose nanofiber composite electroplating are unique in the world. There are no similar studies applying cellulose nanofibers to composite plating other than the studies in our laboratory: the development of Tin (Sn)–copper sulfide (CuS)-supported cellulose nanofiber composite solder foils using the electroplating method was conducted by Kogure and Kobayashi et al. in 2021 [20,21], and the fabrication of Ni–phosphorus (P)–cellulose nanofiber composite-plated films using the electroless plating method was conducted by Kawanabe and Iioka et al. in 2022 [22,23].

It is estimated that approximately 23% of the world’s energy consumption is derived from friction [24]; thus, the development of further wear-resistant coatings is a very important issue to address in order to reduce resource and economic losses [24,25,26]. In the application of Ni–cellulose nanofiber composite electroplated films to wear-resistant coatings, conventional plated films with a high wear resistance and their issues are described, in addition to a comparison with Ni–cellulose nanofiber composite electroplated films and their advantages, as follows:

(1) Ni–silicon carbide (SiC) composite film—The production of SiC ingots is reported to consume 7000 kWh/ton of electricity [27], whereas, for example, cellulose nanofiber production using the 2,2,6,6-tetramethylpiperidine 1-oxyl (TEMPO) oxidation method [28,29,30] is reported to consume 100–500 kWh/ton [31]. Further, since plants, as raw materials, are a renewable resource, it can be said that Ni–cellulose nanofiber composite films have an advantage in terms of sustainability.
(2) Ni–polytetrafluoroethylene (PTFE) composite film—Since PTFE has very strong water repellency, a strong surfactant is required to disperse it in the plating solution. Conventionally, perfluorooctanesulfonic acid (PFOS) and perfluorooctanoic acid (PFOA) have been used for this function, but both are regulated by the Stockholm Convention on Persistent Organic Pollutants (POPs Convention) because they are refractory substances with strong toxicity [32,33]. In its original state, cellulose has hydroxy groups on its surface, making it a hydrophilic substance, and CNF disperses well in the plating solution without the use of special surfactants.
(3) Ni electroless-plated film—Compared to a Ni electroplating solution, a Ni electroless plating solution has a shorter bath life, and it contains phosphate as a reducing agent and prevents bath decomposition, which makes waste solution treatment difficult [34,35,36]. Although the development of plating solutions without lead, which is restricted by the hazardous substances directive (RoHS), and waste solution recycling technologies are in progress, Ni electroplating has an advantage in terms of environmental protection [36]. In addition, in Ni electroless plating, it is more difficult to manage the bath, and it is more expensive to process. Ni electroplating is also easier to apply in developing countries, where facilities are not fully developed.
(4) Hard chromium plating—Hard chromium (Cr) plating is an extremely hard and wear-resistant plated film obtained via electroplating. Its hardness reaches over 800 HV, and it is used for many sliding parts, such as the pistons and cylinder liners of automobile engines, steel-rolling rolls, shafts, and rods [37,38,39]. Hard Cr plating baths have conventionally contained hexavalent chromium, which is restricted by RoHS, and there is an urgent need to replace it. Decorative Cr plating with a trivalent chromium bath, which is not restricted, is widely used, but the hard Cr film obtained from this bath still has many problems in terms of adhesion, workability, wear resistance, etc. [38,39,40].

As described above, Ni–cellulose nanofiber composite electroplated films have many advantages in terms of sustainability and a low environmental load. In our previous studies [1,2,3,4], their fabrication conditions were investigated and revealed; however, their wear resistance has not yet been evaluated. Therefore, in order to evaluate the wear resistance and clarify the abrasion mechanism, the properties of Ni–cellulose nanofiber composite films plated on steels were investigated, and ball-on-disk abrasion tests under non-lubricated (dry) conditions and an analysis of the macro- and micro-structures of abrasion scars were conducted.

## 2. Experimental Procedure

In this study, Ni–cellulose nanofiber composite electroplated films were fabricated on steel plates using a nickel electroplating solution with the addition of cellulose nanofibers. The plating process was mainly based on previous studies [2,3,4], and the range of 1–3 g/L for the addition of cellulose nanofiber was also determined based on our previous study [4]. The obtained plated films were evaluated in terms of surface morphology, quantitative analysis, surface hardness, and Ni crystallite size. The wear resistance of the plated films was evaluated in abrasion tests under dry conditions. In addition, the microstructure of abrasion scars was evaluated using an elemental mapping analysis.

### 2.1. Plating Process

**Figure 1:**
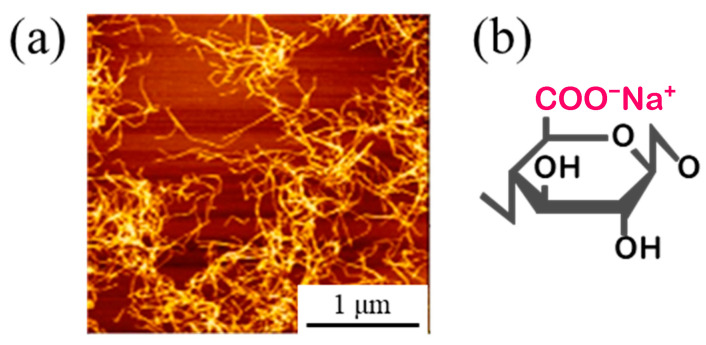
Representative AFM image of CNF (**a**) and its structural formula (**b**) [41].

**Figure 2:**
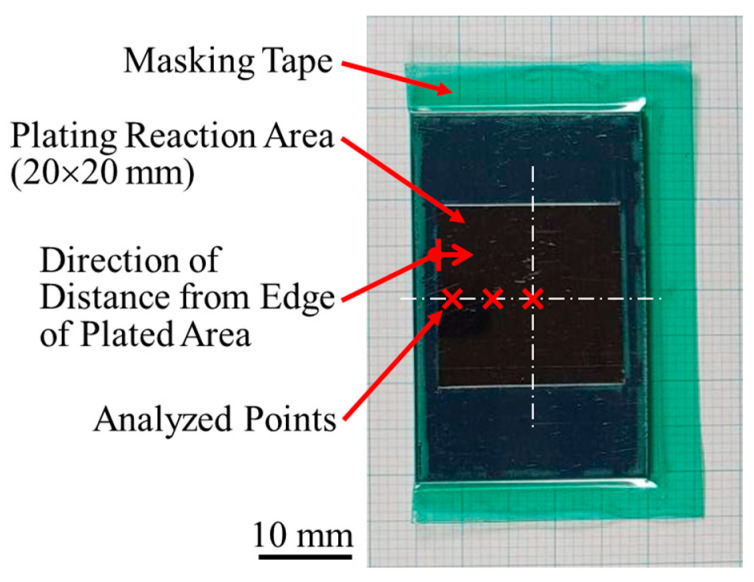
Appearance of plated material and overview of analyzed points. Note that analyzed points shown in this figure do not exactly correspond to actual analyzed points.

**Figure 3:**
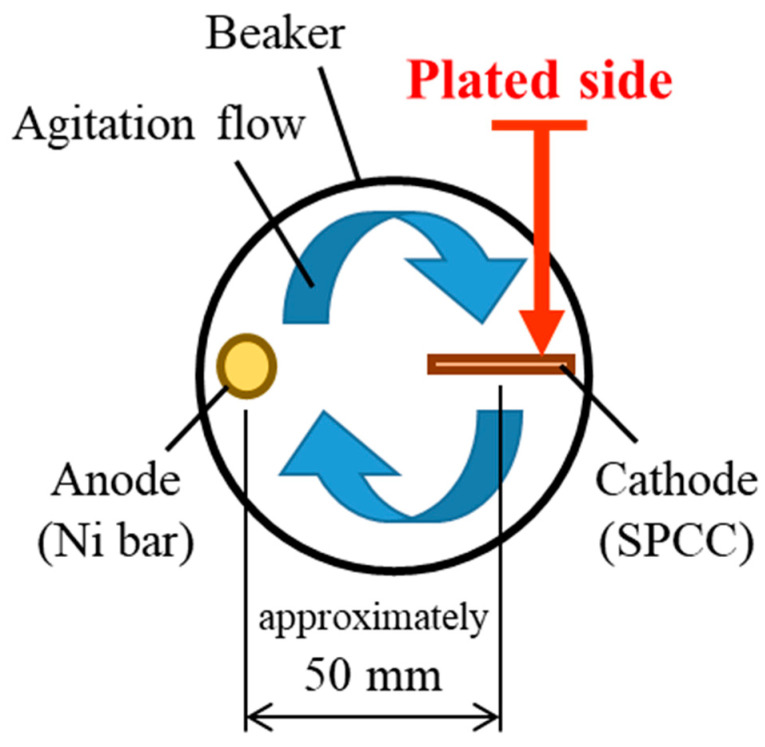
Relationship between placement position of plated material and direction of agitation flow as viewed from top of beaker [2].

A Watts bath of standard composition with the addition of TEMPO-oxidized cellulose nanofibers (TC-01A, Nippon Paper Industries, 1.1 mass% gel, CNF hereinafter) was used as the plating solution, and the composition is shown in Table 1. All reagents used for the plating solution were obtained from FUJIFILM Wako Pure Chemical (Osaka, Japan): nickel(II) sulfate hexahydrate (148-01175, NiSO_4_·6H_2_O, more than 99 mass% purity), nickel(II) chloride (145-01065, NiCl_2_, more than 95 mass% purity), and boric acid (021-02195, H_3_BO_3_, more than 99.5 mass% purity). The representative diameter of CNF was approximately 2–4 nm, according to the manufacturer’s data [41]. Figure 1 shows an atomic force microscopy (AFM) image and the chemical structure of CNF. Steel plates made of JIS SPCC-SB (cold-rolled steel) were used as the plated materials (40 × 25 × 1 mm). The chemical composition of SPCC is shown in Table 2. “SB” indicates surface luster. The plated material was covered with masking tape for plating, as shown in Figure 2, and the plated reaction area was set to 20 × 20 mm. The plated area was then pickled with 30–35 mass% hydrochloric acid and used for plating experiments. Plating experiments were performed in a 300 mL beaker with 250 mL solution at 50 °C under constant agitation with a magnetic stirrer (*ϕ* 8 × 38 mm, 300 rpm) using a DC power supply. Table 3 shows the plating conditions. The current density and current charge density were set to 2.5 A/dm^2^ and 12,000 C/dm^2^, respectively, and the calculated film thickness was approximately 40 µm (refer to Equation (1)). The anode was a Ni rod with a purity higher than 99 mass% (immersed part dimensions *ϕ* 6 × 50 mm), and the distance between electrodes was approximately 50 mm. The surface to be plated, which had a 20 × 20 mm area, was set perpendicular to the agitating flow (refer to Figure 3).

(1) $$d=\frac{\frac{\sigma /F}{n}\;\times \;M}{\rho }\;\times \;\eta$$

d=the thickness of the plated metal (µm);
$\sigma =\mathrm{the}\;\mathrm{charge}\;\mathrm{density}\;(C/{\mathrm{dm}}^{2})$;
$F=\mathrm{the}\;\mathrm{Faraday}\;\mathrm{constant}\;(9.6485\times 10^{4})\;(C/\mathrm{mol})$;
n=the ion valence of the plated metal (-);
M=the molar mass of the plated metal (g/mol);
$\rho =\mathrm{the}\;\mathrm{density}\;\mathrm{of}\;\mathrm{the}\;\mathrm{plated}\;\mathrm{metal}\;(g/{\mathrm{cm}}^{3})$;
η=the cathodic current efficiency (%).

**Table 1:** Composition of plating solution.

	Distilled Water	NiSO_4_·6H_2_O	NiCl_2_	H_3_BO_3_	CNF
(g/L)	Bal.	240	30	30	0–3

**Table 2:** Chemical composition of SPCC.

	Fe	C	Mn	P	S
(mass%)	Bal.	0–0.15	0–0.60	0–0.10	0–0.05

**Table 3:** Plating conditions.

Current Density (A/dm^2^)	Charge Density (C/dm^2^)	Plating Time (min)	Stirring Speed (rpm)
2.5	12,000	80	300

### 2.2. Analysis Process of Plated Films

The following analysis was performed on the analyzed points shown in Figure 2, from the edge to the center along the center line of the plated area.

#### 2.2.1. Observation of Plated Films

Optical and secondary electron (SE) images of the plated film surface were observed. An electron probe X-ray micro-analyzer (EPMA-1610, Shimadzu, EPMA hereinafter) was used to observe the SE images at an acceleration voltage of 15 kV.

#### 2.2.2. Surface Roughness Measurement

The plane roughness of the surfaces of the plated films, the arithmetic mean height, *Sa*, and the maximum height, *Sz* (ISO 25178 [42]), were measured using a laser microscope (VK-X150, Keyence, Osaka, Japan). The measurement area was 1.0 × 1.4 mm.

#### 2.2.3. Evaluation of Dispersion of CNFs on Plated Films

**Figure 4:**
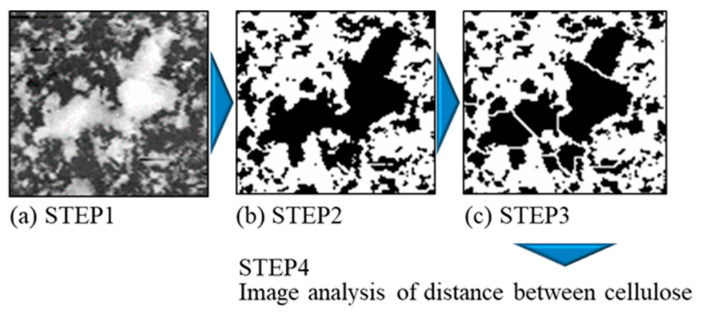
Overview of image analysis procedure of distance between CNFs in plated film. Black and white areas after binarization indicate CNF and Ni parts, respectively. In STEP 1, the prepared composition image is shown (SC image) (**a**); in STEP 2, binarization is performed (**b**); and in STEP 3, watershed treatment is applied (**c**) [2].

The distance between co-deposited CNFs was measured using an image analysis of the specimen current (SC) images obtained via the EPMA of the surfaces of the plated films. ImageJ [43] and its plug-in, ND [44], were used as the software for the image analysis, following the process shown in Figure 4. The obtained SC images were binarized into CNF and Ni parts, and then they were watershed-treated to avoid the perception of overlapping CNFs as huge clusters. The images with these treatments were subjected to image analysis. The distance was defined as the average of the edge-to-edge distance between six neighboring CNF areas. Feret’s diameters of CNF areas were also measured in this process. The measurement area was 1.0 × 1.4 mm. The top 10% values of the distance between CNFs and their Feret’s diameters obtained from the analysis were used for the evaluation.

#### 2.2.4. Quantification Analysis

The EPMA was used to measure the content of CNFs on the surfaces of the plated films. The measured values were obtained as the equivalent content of compound cellulose, (C_6_H_10_O_5_)_n_, by using dried solidified cellulose (nanoforest-S, Chuetsu Pulp & Paper, 10.83 mass% slurry) as a standard sample of 100 mass%C. The quantification analysis was performed at an acceleration voltage of 15 kV and a beam current of 10 nA, and the measurement area was 1.0 × 1.4 mm.

#### 2.2.5. Vickers Hardness Test

The Vickers hardness of the surfaces of the plated films was measured using a Vickers hardness tester (HMV-1 TADW, Shimadzu, Kyoto, Japan). The test load was set to 25 gf, and the test duration was set to 15 s.

#### 2.2.6. X-ray Diffraction Analysis

The crystallite size of the plated film was measured using an X-ray diffractometer (RINT2200VF, Rigaku, Akishima, Japan). A characteristic X-ray of CuKα at a wavelength of 0.15418 nm was used for the analysis. The analysis was conducted with the scanning angle (2*θ*) set to 70–105 degrees, the step width set to 0.02 degrees, the acceleration voltage of the X-ray tube set to 40 kV, and the tube current set to 20 mA. The plated films were used as plated for the analysis, and, due to the characteristics of the equipment, approximately the entire plated area was analyzed in one go. The crystallite size was calculated from the measured X-ray diffraction spectrum using the Scherrer equation shown in Equation (2). The Scherrer constant of 0.94 was used for the calculation. The peak position and spectrum width were defined as the position where the peak top was measured and full width at half maximum (FWHM), respectively.

(2) $$\tau \;=\frac{K\lambda }{\beta \cos\theta }$$

τ =the crystallite size (nm);
K=the Scherrer constant (-);
λ=the wavelength of the X-ray (nm);
β=FWHM (rad);
θ=the Bragg angle (rad).

### 2.3. Abrasion Test

#### 2.3.1. Abrasion Test Using Ball-on-Disk Method

The abrasion tests for the plated films, plated material, SPCC, and SUJ2 disk as a reference material were conducted using a ball-on-disk tribometer (FRP-2100, Rhesca, Hino, Japan). A ball made of JIS SUJ2 and 3/16 inches (4.7625 mm) in diameter was used as the counterpart material. The chemical composition of SUJ2 is shown in Table 4. The average Vickers hardness of the cross-section of the ball was 725 HV. The ball was placed near the center of the plated area with a load of 200 gf and reciprocated at a speed of 10 rpm on a circular arc with a radius of 15 mm and a center angle of 30 degrees. The test duration was 100,000 s (approximately 28 h) at room temperature. The size of the SUJ2 disk was *ϕ* 55 × 5 mm, and it was used for the abrasion tests in both as-received and heat-treated conditions. The average Vickers hardness of the surfaces of the disk was 290 HV as-received and 562 HV with heat treatment. The heat treatment was performed using the following procedures: heating at 850 °C for 150 min, oil quenching, and tempering at 165 °C for 90 min. Abrasion powder was not removed during the test. After the test, the specimens were washed via ultrasonic cleaning with methanol.

#### 2.3.2. Observation and Analysis of Abrasion Scars

##### Abrasion Scars of Plated Films

Optical images of abrasion scars were observed using a laser microscope. The abrasion loss (volume) and the width and depth of the abrasion scars were measured using the function of 3D scanning. Moreover, the EPMA was used to observe the SE images of the abrasion scars and to perform an elemental mapping analysis at an acceleration voltage of 15 kV.

##### Abrasion Scars of Counterpart Balls

**Figure 5:**
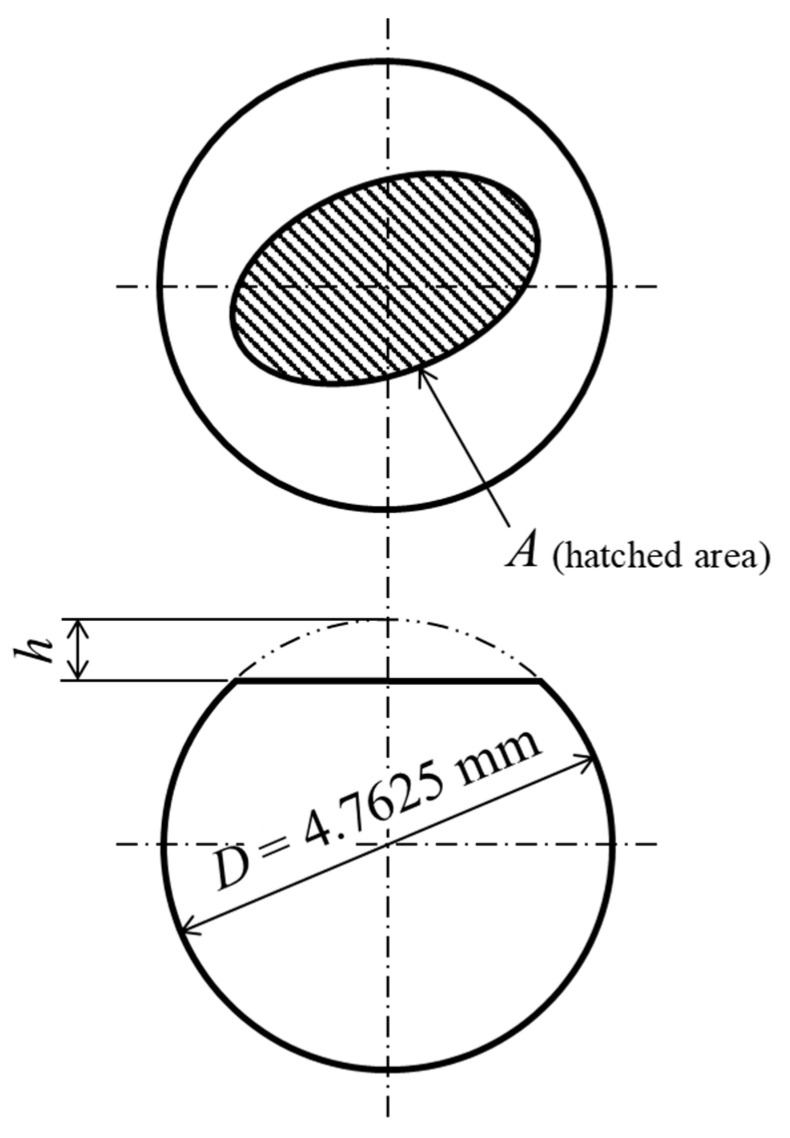
Schematic diagram of abraded counterpart ball.

The optical images of the abrasion scars were observed using a laser microscope. The abrasion loss was calculated using Equations (3)–(5) and, as shown in Figure 5, with values measured via 3D scanning: the horizontal projected area of the abrasion scars and abrasion height.

(3) $$V_{b}=\frac{V_{b,\;A}+V_{b,\;D}}{2}$$

(4) $$V_{b,\;A}=\frac{\pi }{6}h\left(\frac{3}{\pi }A+h^{2}\right)$$

(5) $$V_{b,\;D}=\frac{\pi }{6}h^{2}(3D-2h)$$

$V_{b}=\mathrm{the}\;\mathrm{abrasion}\;\mathrm{loss}\;\mathrm{of}\;\mathrm{the}\;\mathrm{ball}\;({\mathrm{mm}}^{3});$
$V_{b,\;A}=\mathrm{the}\;\mathrm{abrasion}\;\mathrm{loss}\;\mathrm{of}\;\mathrm{the}\;\mathrm{ball}\;\mathrm{calculated}\;\mathrm{based}\;\mathrm{on}\;A\;\mathrm{and}\;h({\mathrm{mm}}^{3});$
$V_{b,\;D}=\mathrm{the}\;\mathrm{abrasion}\;\mathrm{loss}\;\mathrm{of}\;\mathrm{the}\;\mathrm{ball}\;\mathrm{calculated}\;\mathrm{based}\;\mathrm{on}\;D\;\mathrm{and}\;h({\mathrm{mm}}^{3});$
h=the abrasion height (mm);
$A=\mathrm{the}\;\mathrm{horizontal}\;\mathrm{projected}\;\mathrm{area}\;\mathrm{of}\;\mathrm{the}\;\mathrm{abrasion}\;\mathrm{scars}\;({\mathrm{mm}}^{2});$
D=the diameter of the ball (mm) (4.7625 mm).

## 3. Results and Discussion

### 3.1. Properties of Plated Films

#### 3.1.1. Morphology and Microstructure

**Figure 6:**
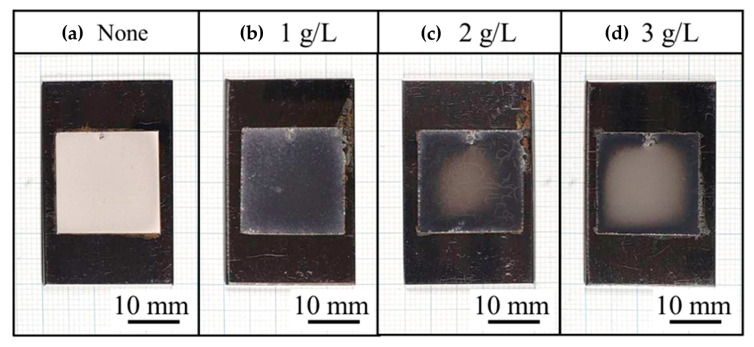
Appearance of Ni-plated films from conventional Watts bath solution (**a**) and solution with addition of CNF at 1 g/L (**b**), 2 g/L (**c**), and 3 g/L (**d**).

Figure 6 shows the appearance of the plated films. As in previous studies [2,3,4], the plated film without CNF was matte, and the plated film with CNF was bright, while the plated film with CNF showed uneven brightness under the conditions where 2 and 3 g/L of CNF were added.

**Figure 7:**
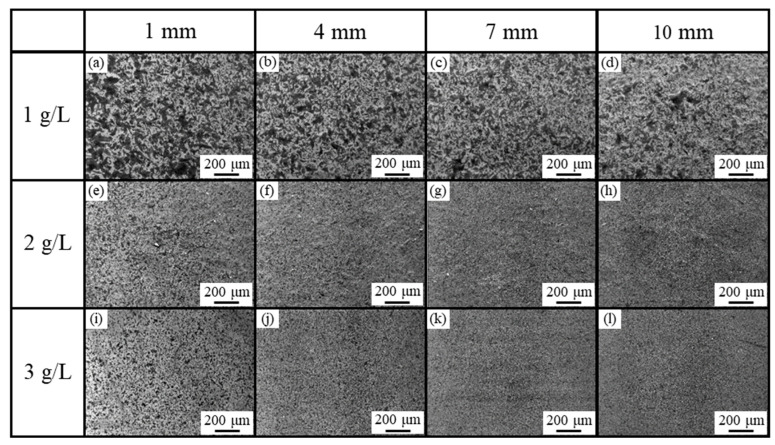
SE images of surfaces of plated films obtained from solution with CNF addition at 1 g/L (**a**–**d**), 2 g/L (**e**–**h**), and 3 g/L (**i**–**l**). Values in top row indicate distance from edge of plated area.

Figure 7 shows the SE images of the surfaces of the plated films with CNF and dark gray areas where CNFs co-deposited were observed, whereas the plated film with 1 g/L of CNF seemed to have slightly more aggregated CNFs co-deposited than the plated films with 2 or 3 g/L. This is a different trend from our previous study [4], which showed that the higher the CNF addition, the more aggregated the CNFs. Although a little aggregation of CNFs was observed near the edge of the plating area regardless of the amount of CNF added, no significant differences in microstructure depending on the position were observed. Generally, in electroplating, the non-uniformity of the electric field distribution and agitation causes a slight unevenness in the film state.

**Figure 8:**
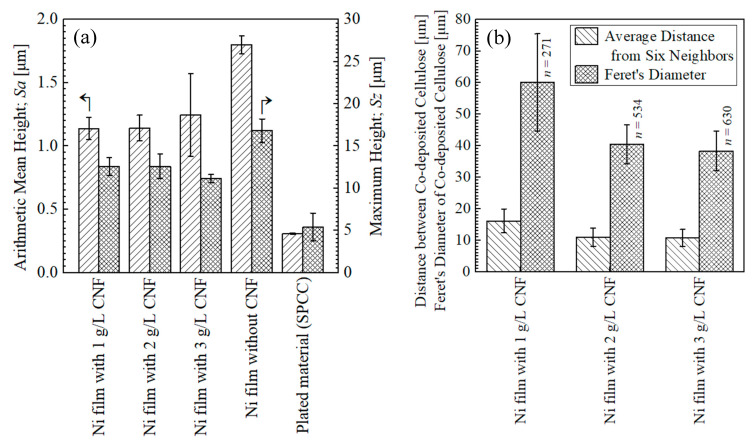
Surface roughness of plated films and as-received SPCC measured at 1, 5.5, and 10 mm from edge of plated area (**a**) and distance between co-deposited CNFs and their Feret′s diameter of co-deposited CNFs on surfaces of plated films measured at 1, 4, 7, and 10 mm from edge of plated area (**b**). *n* indicates the top 10% number of sites recognized as CNF.

Figure 8a shows the surface roughness of the plated films and SPCC. The SPCC-SB steel plates used in this study had a mirror-like luster in the as-received state, and the measured surface roughness was very small, approximately 0.3 µm in *Sa*. The plated films with CNF showed approximately 1.2 µm in *Sa*, and the plated film without CNF showed approximately 1.8 µm in *Sa.* The grain refinement effect due to Ni deposition from the chelate complex between the Ni ions and carboxylates on CNF made the surface roughness smooth [2].

Figure 8b shows the distance between the co-deposited CNFs and their Feret’s diameters. The distance between the co-deposited CNFs averaged 16–10 µm with the addition of 1–3 g/L of CNF. The Feret’s diameters with the addition of 1 g/L of CNF were approximately 60 µm, larger than those with the addition of 2–3 g/L of CNF by approximately 20 µm, which quantitatively represents the higher degree of aggregation with the addition of 1 g/L of CNF, as shown in Figure 7.

#### 3.1.2. Content of Cellulose

**Figure 9:**
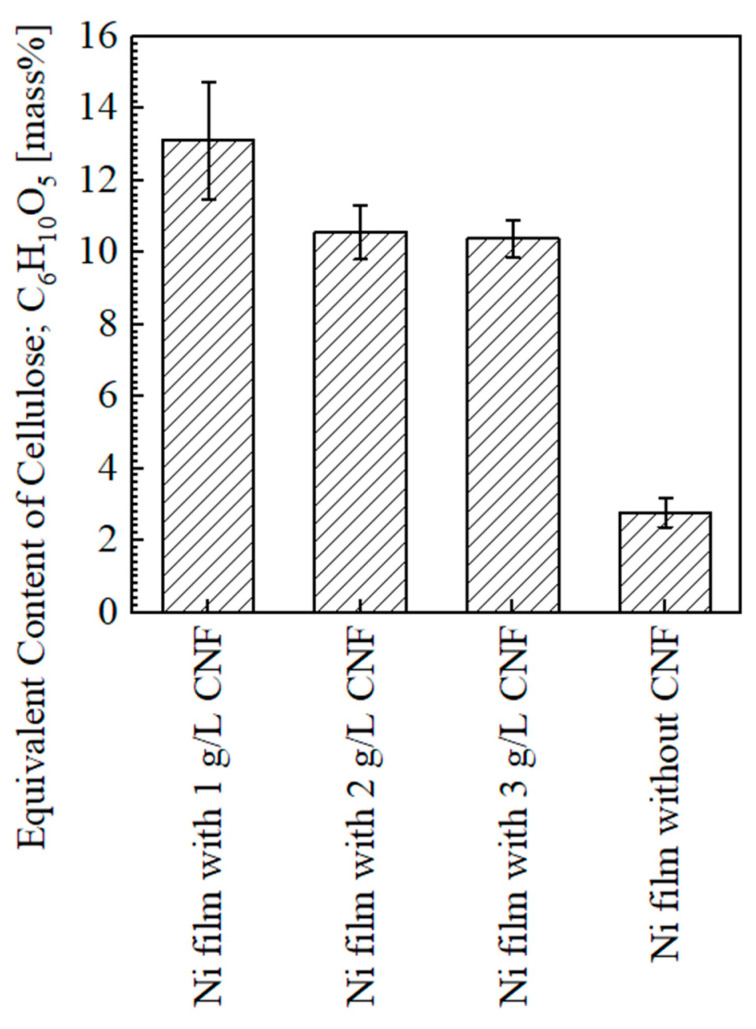
Results of quantification analysis of cellulose equivalent on surfaces of plated films measured at 1, 4, 7, 10 mm from edge of plated area for Ni films with CNF, and 1, 5.5, 10 mm for Ni films without CNF.

Figure 9 shows the equivalent content of cellulose on the surfaces of the plated films. The average content of CNF on the plated film with the addition of 1 g/L of CNF was approximately 13.1 mass% and the highest in this study. The content with the addition of 2 and 3 g/L of CNF was approximately 10.6 and 10.4 mass%, respectively (a similar degree). The detection of several mass% values on the plated film without CNF was considered to be background noise [2]. In our previous study [4], the content of cellulose showed an increasing trend with the increase in CNF in the range of 1–3 g/L; thus, the trend in this study is different from that.

The reason for this is assumed to be the changes in the agitation state of the solution due to the changes in the size of the plated material and the size of the beaker used as the plating bath. Although the stirrer size and stirring speed were the same, the plated material size in the previous study was 10 × 10 × 0.3 mm, and a 200 mL beaker with a 200 mL solution volume was used as the bath [4], while in this study, the plated material size was 40 × 25 × 1 mm, and a 300 mL beaker with a 250 mL solution volume was used as the bath. It is considered that this caused insufficient agitation. In our previous study [4], it was reported that the cellulose content on the plated film surface decreased due to the aggregation of CNFs in the bath when the amount of CNF added was 4–5 g/L. In the case of this study, aggregation occurred even when a smaller amount of CNF was added due to insufficient agitation, and, at the same time, the agitation flow was not sufficient to transport the aggregated CNFs to the surface of the plated material. This probably led to the result where the content of cellulose in the plated film decreased as the amount of CNF added increased. However, under the condition of 1 g/L CNF addition, the content of cellulose was supposed to increase due to the co-deposition of CNF aggregates in the plated film, which were small enough to be carried, even by a weak agitation flow.

#### 3.1.3. Hardness of Surfaces and Crystallite Size

**Figure 10:**
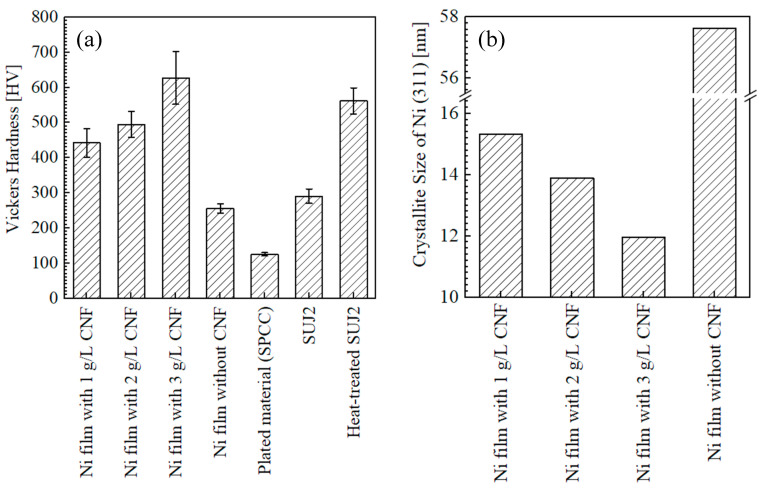
Vickers hardness of surfaces of plated films, as-received SPCC, and SUJ2 disks (**a**) and crystallite size of Ni (311) of plated films calculated using Scherrer equation (**b**). Hardness of films and SPCC was measured at 1–9 mm from edge of plated area in steps of 1 mm. No significant change in hardness was observed depending on measurement position.

Figure 10a shows the Vickers hardness of the surfaces of the plated films and SPCC. In contrast to the trend in the content of cellulose, the change in Vickers hardness with respect to the amount of CNF added was similar to the trend in our previous study [4], with an increase in the Vickers hardness with an incremental addition of CNF in the range of 1–3 g/L. The hardness values of the plated films with the addition of 1, 2, and 3 g/L of CNF were, on average, 443, 495, and 627 HV, respectively. The hardness value of the plated film with 3 g/L of CNF at 627 HV was higher than that of the heat-treated SUJ2 at 562 HV. For the reasons underlying the hardness improvement, dispersion enhancement due to the fine dispersion of the co-deposited CNFs and the increase in the cellulose content was considered [2,4]. However, in this experiment, the plated film with 3 g/L of CNF and the highest hardness showed the lowest cellulose content. Thus, the crystallite size of electroplated Ni, calculated from the X-ray diffraction spectrum, was evaluated next.

Figure 10b shows the crystallite size of Ni (311) calculated from the X-ray diffraction spectrum using the Scherrer equation (Equation (2)). Since the crystallite size decreases with increasing CNF addition, it can be inferred that the increase in hardness with increasing CNF addition is due to crystallite refinement, in addition to dispersion enhancement. However, note that the Scherrer equation is a simple method for calculating the crystallite size, so the calculated crystallite size is used for a relative, not absolute, evaluation [45].

It has been considered that nickel is deposited randomly and in a non-oriented manner from the chelate complex formed between the carboxylate of CNF and Ni ions, and this causes CNFs to be incorporated into the Ni-plated film, which leads to the grain refinement and dispersion enhancement effects, as the mechanisms of Ni–CNF composite electroplating [2,46]. According to that, it is necessary for nickel to be deposited via chelate complexes between the carboxylate of CNF and Ni ions in order for the crystal grains to become finer. In addition, crystal nucleation should be promoted by fine CNF co-deposition [2]. Previously, in Section 3.1.2, it was stated that the aggregated CNFs in the plating solution were not transported to the surface of the plated material; however, it was possible that fine CNFs sufficiently reached the surface of the plated material. Although the degree of aggregation that occurs in the solution when the amount of CNF added increases was not verified in this study, if the amount of fine CNFs that escaped aggregation increased in the solution when the amount of CNF added increased, the amount of Ni ion-coordinated fine CNF transported to the plated material surface could also be considered to have increased. As a result, it was considered possible that the crystallite size refinement and hardness increase associated with the increase in CNF addition occurred under the influence of Ni deposition via chelate complexes and fine CNF co-deposition.

Naturally, if a lot of fine CNFs are co-deposited, the cellulose content of the plated film also increases. Therefore, it is not wrong to consider that a higher cellulose content of the plated film increases the hardness of the plated film if the degree of dispersion is the same. Previous studies examined a plated film with sodium carboxymethyl cellulose (CMC), which had large-diameter co-deposited sites exposed on the plated surface and a high cellulose content detected using the same quantification method in the EPMA as in this study [2,3]. As in this case, when the co-deposition of large-diameter cellulose fibers or aggregated CNFs on the surface is measured, the content of cellulose tends to be higher than the actual content of the whole film due to the characteristics of the quantitative analysis of the surface using the EPMA [2]. Therefore, this is likely the reason why the films with the addition of 2–3 g/L of CNF, in which few aggregations were observed on the surfaces, showed a lower cellulose content and a higher hardness than the film with the addition of 1 g/L of CNF.

### 3.2. Wear Resistance of Plated Films

#### 3.2.1. Macrostructure of Abrasion Scars

**Figure 11:**
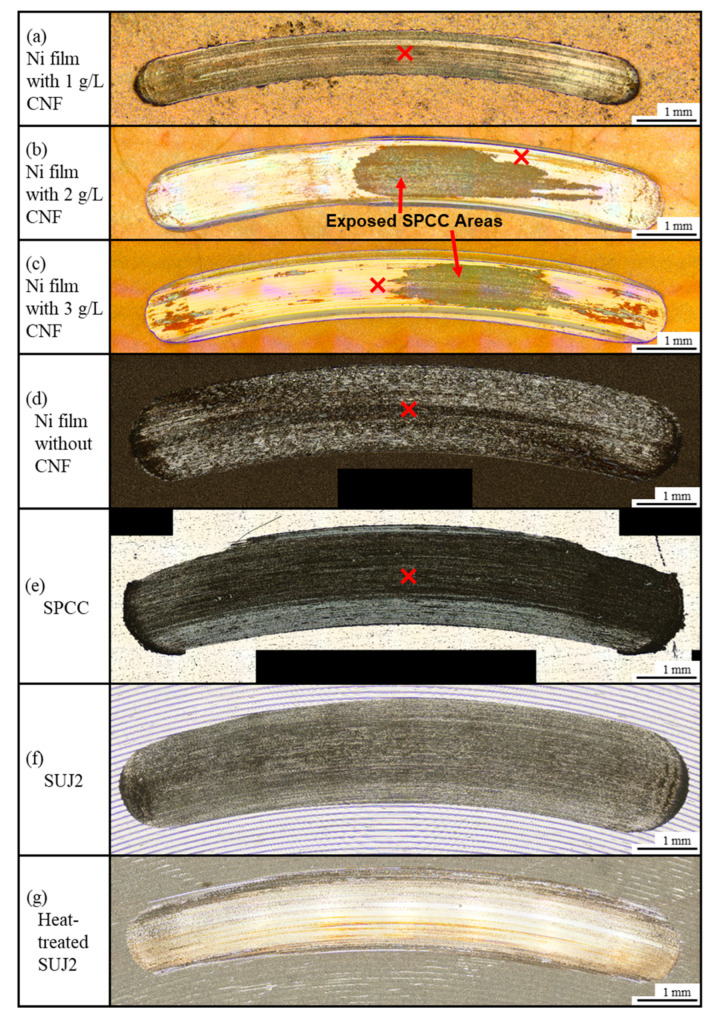
Representative appearance of abrasion scars of plated films, SPCC, and SUJ2 disks. ✕ marks indicate points of observation of microstructure of abrasion scars.

**Figure 12:**
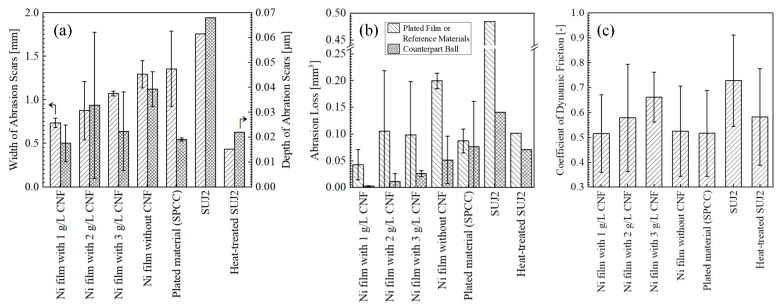
Maximum width and depth of abrasion scars (**a**), abrasion losses (**b**), and coefficient of dynamic friction averaged at 50,000–53,600 s (**c**) of plated films (*n* = 2), SPCC (*n* = 2), and SUJ2 disks (*n* = 1).

Figure 11 shows the appearance of abrasion scars on the plated films and SPCC. Compared to the abrasion scar of the plated film with 1 g/L of CNF, the films with 2 g/L and 3 g/L of CNF showed abrasion scars with larger widths, and the SPCC (the plated material) was exposed. The abrasion scars of the film without CNF, SPCC, and the as-received SUJ2 appeared to be even larger in width. Figure 12a shows the width and depth of the abrasion scars. As shown in the appearance, the averaged maximum width of the abrasion scars of the film with 1 g/L of CNF was the smallest out of all the plated films at approximately 0.74 mm, and that of the abrasion scars of SUJ2 was the largest out of all the specimens at approximately 1.76 mm. However, the width of the abrasion scar of the heat-treated SUJ2 was smaller than that of the abrasion scar of the film with 1 g/L of CNF. The depth of the abrasion scars of the film with 1 g/L of CNF was the shallowest at approximately 0.018 mm, and that of the abrasion scars of SUJ2 was the deepest at approximately 0.068 mm.

**Figure 13:**
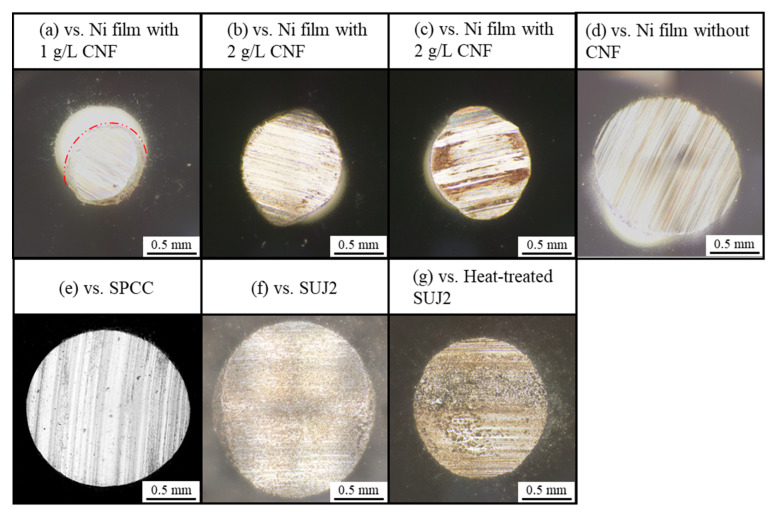
Representative appearance of abrasion scars of counterpart balls.

Figure 13 shows the appearance of the abrasion scars of the counterpart ball. The horizontal projected area of the abrasion scars (hereinafter simply referred to as area) was the smallest for the counterpart ball with the film containing 1g/L of CNF, which had the smallest abrasion scar width on the plated side, while the counterpart ball with SUJ2, which had the largest width, had the largest area.

Figure 12b shows the abrasion losses on the plated sides and on the counterpart balls. The plated film with 1 g/L of CNF had the lowest abrasion losses on the plated side and on the counterpart ball. Compared to the plated film without CNF, the abrasion loss on the plated side and counterpart ball was reduced by approximately 79% and 94%, respectively. Compared to the heat-treated SUJ2, which had the highest hardness, the amount of abrasion on the plated side and the ball side was reduced by approximately 58% and 96%, respectively. The plated films with 2 g/L and 3 g/L of CNF showed approximately half the average amount of abrasion loss on the plated side as the film without CNF; however, they had large errors. Likewise, the abrasion loss of SPCC and the heat-treated SUJ2 was approximately half that of the film without CNF, although the counterpart ball was more abraded. The as-received SUJ2 had the highest amount of abrasion out of all the specimens, both on the disk and counterpart ball sides. Figure 12c shows the coefficient of dynamic friction averaged at 50,000–53,600 s. The plated film with 1 g/L of CNF, which had the lowest abrasion loss, had the lowest coefficient of dynamic friction at approximately 0.52. This suggests that the intervention of CNFs at the abrasion interface possibly caused slippage. In order to determine the mechanism behind the effect of CNF addition on abrasion loss reduction, a microstructural observation and analysis of the wear scars were conducted.

#### 3.2.2. Microstructure of Abrasion Scars

**Figure 14:**
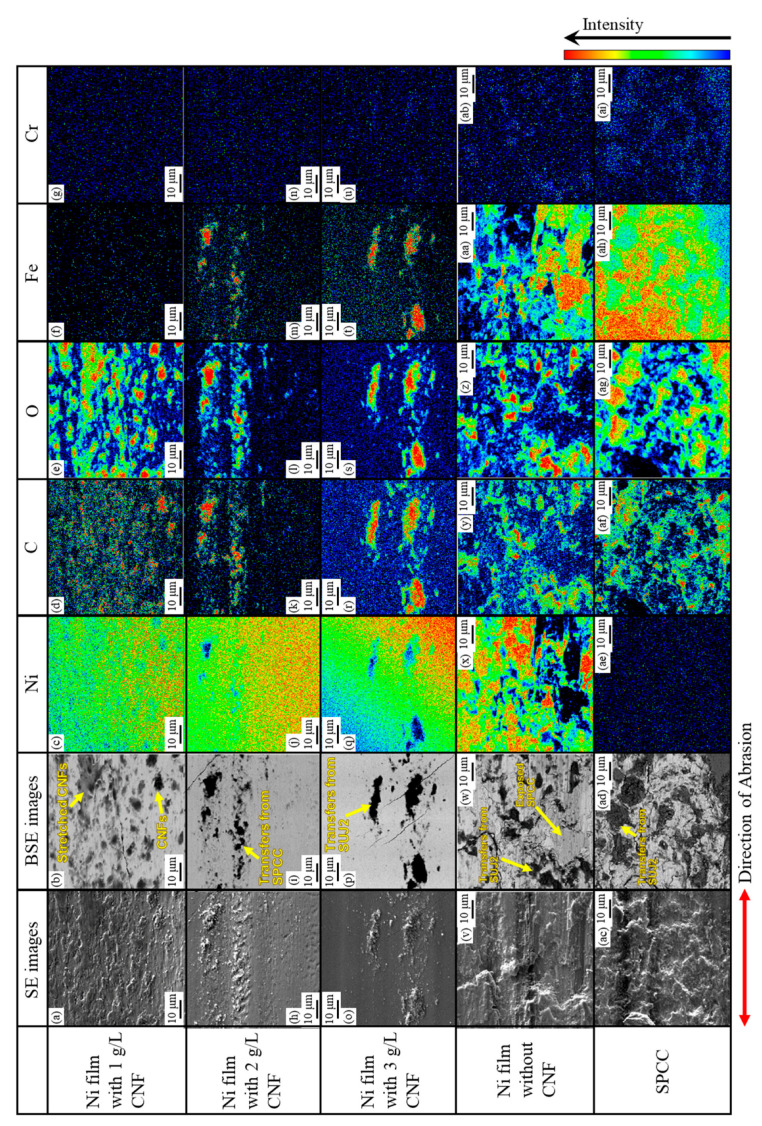
Microstructures of abrasion scars including SE, BSE, and elemental mapping images of Ni-plated film with 1 g/L CNF (**a**–**g**), 2 g/L CNF (**h**–**n**), and 3 g/L CNF (**o**–**u**); without CNF (**v**–**z**,**aa**,**ab**); and SPCC (**ac**–**ai**).

Figure 14 shows the microstructures of the abrasion scars on the plated side, including SE and BSE images and elemental mapping images (nickel, carbon, oxygen, iron, and chromium). For the plated films with the addition of 2 g/L and 3 g/L of CNF, the Ni-plated areas where SPCC was not exposed were observed (refer to Figure 11b,c).

In the abrasion scar of the plated film with 1 g/L of CNF, dark gray areas in the BSE image containing carbon and oxygen were observed, and these were considered to be co-deposited CNFs. Some CNFs were found to be stretched in the direction of abrasion. No iron or chromium was detected, and there was no adhesion or transfer from the substrate SPCC or counterpart ball.

The black areas consisting of carbon, oxygen, and iron shown in the BSE image of the plated film with 2 g/L of CNF were assumed to have been transferred from the substrate SPCC. They were considered to have been transferred from the exposed SPCC via counterparts or abrasion powder, and no co-deposited CNFs were observed.

In the BSE image of the plated film with 3 g/L of CNF, the black areas consisting of carbon, oxygen, iron, and a little chromium were probably transferred from the counterpart ball and SUJ2, and no co-deposited CNFs were observed.

Similarly, the black areas with carbon, oxygen, iron, and a little chromium shown in the BSE image of the plated film without CNF appeared to have been adsorbed from the counterpart balls. The dark gray areas of this BSE image contained mainly iron, confirming that the substrate SPCC was one step away from being fully exposed. The dark gray area shown in the BSE image of the abrasion scar on SPCC was also accompanied by carbon, oxygen, iron, and some chromium, as before, and these were assumed to have been transferred from the counterpart ball. The number of such transfers appeared to be the highest compared to the other abrasion scars.

#### 3.2.3. Abrasion Mechanism

##### Ni–CNF Composite Films

**Figure 15:**
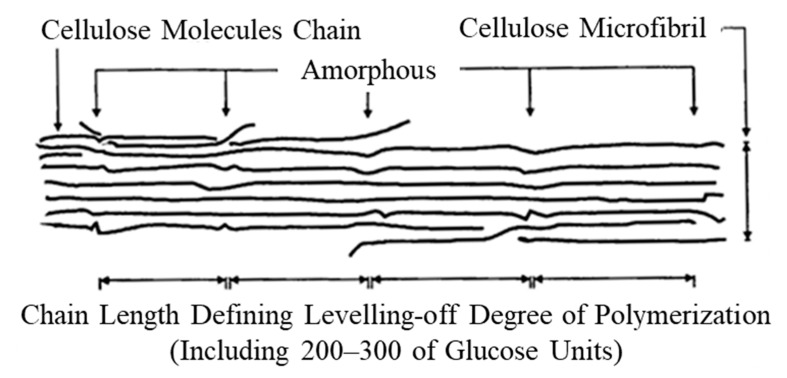
Structure of cellulose microfibril. Leveling-off degree of polymerization is degree of polymerization that becomes constant when natural cellulose fibers are hydrolyzed with dilute acid [48].

In the abrasion scar of the plated film with 1 g/L of CNF, co-deposited CNF aggregates with a diameter of approximately several µm and areas that appeared to be stretched in the direction of abrasion were observed (Figure 14b). Based on these results, it was considered that the friction reduction mechanism may be caused by the sliding deformation of individual CNFs in the aggregates. For example, PTFE, which exhibits an extremely low coefficient of friction and self-lubricating properties, is believed to exhibit a low coefficient of friction due to sliding between molecular chains or between crystalline and amorphous portions [47]. Figure 15 shows the structure of cellulose microfibril (CMF) with a width of approximately 3 nm, which is the smallest unit of CNF. In CMFs, cellulose molecular chains are oriented in the longitudinal direction to form crystalline material with partially amorphous portions, but molecular chains are strongly hydrogen-bonded to each other [48,49]; thus, it is unlikely that the molecular chains slide against each other. It is possible that the deformation of the amorphous part or the deformation of the crystalline part via the amorphous part may cause microscopic slippage; however, it is difficult to verify this possibility, and thus, it is not mentioned much. Returning to the micron order, individual CNFs or CMFs inside the aggregated sites of CNFs are considered to be fibrillated by the TEMPO oxidation process [50] and held together by intermolecular forces or loose physical intertwining between them. Therefore, they are easily deformable, and it is suggested that the abrasion reduction effect may have been exhibited by the aggregate CNFs intervening at the interface of the frictional phenomena, causing slippage.

Based on the above, it is considered that the plated film with the addition of 1 g/L of CNF, which had many aggregated CNF sites on the surface, exhibited a significant friction reduction effect in this study. For the plated films with the addition of 2 g/L and 3 g/L of CNF, the CNFs could not be observed in the abrasion scars, and it is assumed that the insufficient cellulose content did not provide a sufficient abrasion reduction effect.

##### Ni Film and SPCC

**Figure 16:**
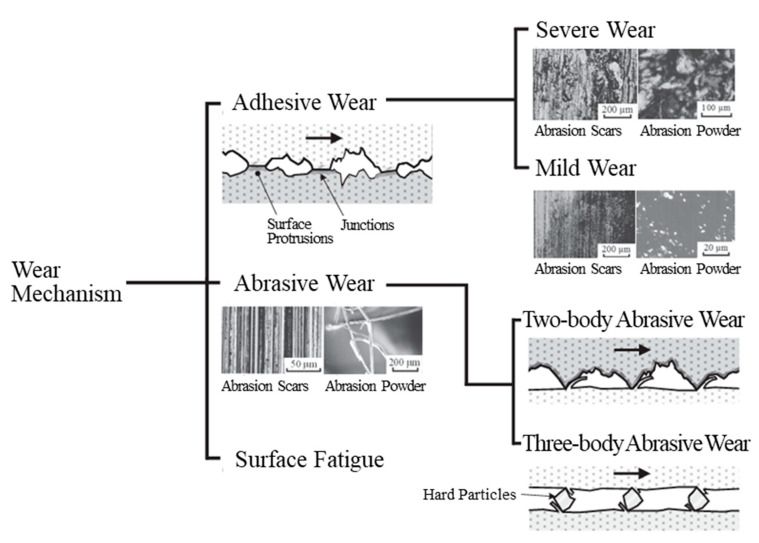
Classification of wear mechanism [51].

The abrasion scars of the plated film without CNF and SPCC appear to be typical of severe adhesive wear with fracture sites where the surface appears to have been torn off [51]. Figure 16 shows the classification of the wear mechanism. Hase presented the experimental results of Mishina et al. in pure metal-to-metal abrasion tests, showing that the combination of iron and nickel resulted in severe adhesive wear, while the combination of iron and iron resulted in a transition from severe to mild abrasive wear with less abrasion loss [51]. In other words, adhesion was higher for the iron–nickel combination, which probably led to the result where the Ni film without CNF was worn more than SPCC in this experiment. Kikuchi et al. showed that pin-on-disk abrasion tests between steels (JIS S45C) resulted in approximately twice the abrasion loss on the pin side as on the disk side by severe adhesive wear [52]. This trend is similar to the abrasion test results of the SUJ2-ball/SPCC combination in this study, in which the ball side’s abrasion ratio was higher than the disk side’s abrasion loss when compared with other combinations. However, since the hardness of the SUJ2-ball averaged at 725 HV, which is higher than that of SPCC, at approximately 125 HV, it is considered that two-body abrasive wear of SPCC via the ball occurred at the same time [53] and resulted in smaller differences in the abrasion losses than in the experiment by Kikuchi et al. [52].

### 3.3. Future Prospects

Since the Ni–CNF composite electroplated film exhibited a high hardness and wear resistance due to the incorporation of CNF, it is expected to be applied to sliding parts, dies, and structural parts in general that require a high hardness or wear resistance. Specifically, in the future, the Ni–CNF composite film is expected to be applied to the following: (1) precision sliding parts for which lubricants such as grease are unsuitable in the fields of aerospace, electronics, and industrial equipment in clean environments and (2) dies used for press work without press oil or phosphate treatment from the perspective of reducing processing and cleaning costs and environmental load [54,55,56] due to the excellent wear resistance of the film under dry conditions, as revealed in this study. In addition, the deposition of the Ni–CNF composite film does not require the use of environmentally restricted substances, such as those regulated by the POPs Convention and RoHS, and CNF itself is a renewable resource, which means that the active use of this film has the advantage of being recommended in accordance with the Sustainable Development Goals (SDGs). Therefore, it is also expected to be used in countries or regions with strict environmental regulations.

## 4. Limitations of This Study

In this study, the Ni–CNF composite electroplated film achieved a significant wear reduction effect and reduced abrasion loss by 94% for the counterpart material compared to the conventional Ni electroplated film. However, there are several limitations to this study, as described in the next paragraph.

The relationship between the amount of CNF added to the plating solution and the cellulose content of the plated films in this study is in contrast to that in our previous study [4]. Therefore, it should be noted that the wear resistance of the plated films obtained in this study should not be understood as a function of the addition of CNF. Moreover, as explained in Section 3.1.3, the pseudo-cellulose quantification method using an elemental analysis of the surfaces of the plated films with the EPMA is not necessarily useful to evaluate the cellulose content of plated films as a whole since the information is obtained only from the surface and is easily affected by the surface morphology. As described so far, it is believed that the abrasion loss is affected by the degree of aggregation of CNF on the plating film and its content according to the abrasion mechanism of the Ni–CNF composite films inferred in this study. Therefore, further investigation of plating conditions, such as the composition of the plating solution and agitation, is necessary to control these parameters.

## 5. Conclusions

In this study, abrasion tests were conducted on Ni–CNF composite electroplated films against steel balls (JIS SUJ2), and a microstructural observation and analysis of abrasion scars were conducted to evaluate the wear resistance of the films. In addition, the crystallite size of the plated films was measured using an X-ray diffractometer. As a result, the following conclusions were obtained:

(1) The Ni–CNF plated films obtained with the addition of 1 g/L of CNF, a current density of 2.5 A/dm^2^, and a stirring speed of 300 rpm exhibited excellent wear resistance. Compared to the conventional Ni-plated film without CNF, the abrasion losses on the plated side and the ball side were reduced by approximately 79% and 94%, respectively;
(2) The mechanism of abrasion reduction by the composite CNFs was considered to be the result of CNF aggregates intervening at the abrasion interface and the individual CNFs within the aggregates sliding;
(3) The Vickers hardness of the surface of the plated films increased as the amount of CNF added to the plating solution increased in the range of 1–3 g/L. Moreover, the Ni (311) crystallite size was confirmed to become finer as the hardness increased;
(4) In contrast to the results in our previous study [4], the content of cellulose in the plated films showed a decreasing trend with the addition of CNF in the range of 1–3 g/L to the plating solution. Insufficient agitation was considered to be the reason for this; however, it was inferred that even weak agitation was not a problem for transporting fine CNFs, leading to crystallite size refinement.

## Figures and Tables

**Table 4:** Chemical composition of SUJ2.

	Fe	C	Si	Mn	P	S	Cr
(mass%)	Bal.	0.95–1.10	0.15–0.35	0–0.50	0–0.025	0–0.025	1.30–1.60

## Data Availability

Raw data were generated at Gunma University. Derived data supporting the results of this study are available upon request from the corresponding author, M.I.

## References

[1] Iioka M., Kawanabe W., Shohji I., Kobayashi T. (2022). An experimental study of fabrication of cellulose nano-fiber composited Ni film by electroplating. Mater. Trans..

[2] Iioka M., Kawanabe W., Kobayashi T., Shohji I., Sakamoto K. (2023). Fabrication of electroplated nickel composite films using cellulose nanofibers introduced with carboxy groups as co-deposited materials. Surfaces.

[3] Iioka M., Kawanabe W., Kobayashi T., Shohji I. (2023). Application of metal sputtering treatment to cellulose materials used as co-deposited material for electrolytic nickel composite plating. Q. J. Jpn. Weld. Soc..

[4] Iioka M., Kawanabe W., Kobayashi T., Shohji I. (2023). Investigation of effects of electroplating conditions on TEMPO-oxidized cellulose nanofiber composited nickel electroplated films. Mater. Sci. Forum.

[5] Yano H. (2016). Cellulose nanofibers and their utilization. J. Imaging Soc. Jpn..

[6] Yano H. (2008). III: Cellulosic nanocomposites. J. Soc. Mater. Sci. Jpn..

[7] Lee K., Aitomäk Y., Berglund L.A., Oksman K., Bismarck A. (2014). On the use of nanocellulose as reinforcement in polymer matrix composites. Compos. Sci. Technol..

[8] Norrrahim M.N.F., Kasim N.A.M., Knight V.F., Halim N.A., Shah N.A.A., Noor S.A.M., Jamal S.H., Ong K.K., Yunus W.M.Z.W., Farid M.A.A. (2021). Performance evaluation of cellulose nanofiber reinforced polymer composites. Funct. Compos. Struct..

[9] Moon D., Tsukahara K., Sagisaka M., Tahara K. (2016). Effect of cellulose nanofibers composites in automotive components on greenhouse gas emissions. J. Jpn. Inst. Energy.

[10] Savadekar N.R., Mhaske S.T. (2012). Synthesis of nano cellulose fibers and effect on thermoplastics starch-based films. Carbohydr. Polym..

[11] Cherian B.M., Leão A.L., Souza S.F.D., Costa L.M.M., Olyveira G.M.D., Kottaisamy M., Nagarajan E.R., Thomas S. (2011). Cellulose nanocomposites with nanofibres isolated from pineapple leaf fibers for medical applications. Carbohydr. Polym..

[12] Fujisaki Y., Koga H., Nakajima Y., Nakata M., Tsuji H., Yamamoto T., Kurita T., Nogi M., Shimidzu N. (2013). Transparent nanopaper-based flexible organic thin-film transistor array. Adv. Funct. Mater..

[13] Koga H., Nogi M., Isogai A. (2017). Ionic liquid mediated dispersion and support of functional molecules on cellulose fibers for stimuli-responsive chromic paper devices. ACS Appl. Mater. Interfaces.

[14] Kang Y.J., Chun S.J., Lee S.S., Kim B.Y., Kim J.H., Chung H., Lee S.Y., Kim W. (2012). All-solid-state flexible supercapacitors fabricated with bacterial nanocellulose papers, carbon nanotubes, and ion gels. ACS Nano.

[15] Nogi M., Iwamoto S., Nakagaito A.N., Yano H. (2009). Optically transparent nanofiber paper. Adv. Mater..

[16] Rocha J.H.A., Farias L.D.N., Siqueira T.P.L. (2022). Cellulose nanofibers (CNF) as reinforcement for cementitious matrices: A systematic literature review. Rev. ALCONPAT.

[17] Subramanian R., Hiltunen E., Gane P.A.C., Kalia S., Kaith B.S., Kaur I. (2011). Potential Use of Micro- and Nanofibrillated Cellulose Composites Exemplified by Paper. Cellulose Fibers: Bio- and Nano-Polymer Composites.

[18] Kurita H., Kanno T., Narita F. (2022). Progress on materials reinforcement using mechanically defibrillated cellulose nanofibers. J. Soc. Mater. Sci. Jpn..

[19] Osada T., Kobayashi S. (2023). Mechanical properties and deformation behavior of metal injection molded products with cellulose nanofiber. J. Jpn. Soc. Powder Powder Metall..

[20] Kogure A., Shohji I., Kobayashi T. Fabrication of lead-free solder by Sn–cellulose nanofiber composite plating. Proceedings of the 28th Symposium on Microjoining and Assembly Technology in Electronics (Mate 2022).

[21] Kobayashi T., Kogure A., Shohji I. Development of Sn solder plating containing cellulose nanofiber. Proceedings of the 2022 International Conference on Electronics Packaging (ICEP 2022).

[22] Kawanabe W., Iioka M., Shohji I., Kobayashi T. Investigation of compositing method of cellulose nano-fiber into nickel plating film by electroless method. Proceedings of the 29th Symposium on Microjoining and Assembly Technology in Electronics (Mate 2023).

[23] Kawanabe W., Iioka M., Kobayashi T., Shohji I. (2023). Investigation of deposition conditions and basic properties of CNF composite Ni plated film by electroless plating method. Mater. Sci. Forum.

[24] Holmberg K., Erdemir A. (2017). Influence of tribology on global energy consumption, costs and emissions. Friction.

[25] Gu Y., Xia K., Wu D., Mou J., Zheng S. (2020). Technical characteristics and wear-resistant mechanism of nano coatings: A review. Coatings.

[26] Zaki E.G., Selim M.S., Hao Z., Elsaeed S.M., El-Saeed A.M. (2023). Special issue: Recent trends in wear and erosion resistance of alloys. Coatings.

[27] Tanaka H. (2011). Silicon carbide powder and sintered materials. J. Ceram. Soc. Jpn..

[28] Saito T., Isogai A. (2004). TEMPO-mediated oxidation of native cellulose. The effect of oxidation conditions on chemical and crystal structures of the water-insoluble fractions. Biomacromolecules.

[29] Saito T., Isogai A. (2005). Ion-exchange behavior of carboxylate groups in fibrous cellulose oxidized by the TEMPO-mediated system. Carbohydr. Polym..

[30] Saito T., Okita Y., Nge T.T., Sugiyama J., Isogai A. (2006). TEMPO-mediated oxidation of native cellulose. Microscopic analysis of fibrous fractions in the oxidized products. Carbohydr. Polym..

[31] Petroudy S.R.D., Chabot B., Loranger E., Naebe M., Shojaeiarani J., Gharehkhani S., Ahvazi B., Hu J., Thomas S. (2021). Recent advances in cellulose nanofibers preparation through energy-efficient approaches: A review. Energies.

[32] Sato M. (2009). Hydrophobic process by composite plating. J. Surf. Finish. Soc. Jpn..

[33] Nishikawa K. (2015). Electroless Ni–P/PTFE composite plating. J. Surf. Finish. Soc. Jpn..

[34] Sugizaki T. (2006). Environment conscious electroless Ni–P plating. J. Surf. Finish. Soc. Jpn..

[35] Hashizume K., Naito K., Oka H., Okumura H. (2007). The situation and future of electroless nickel plating. J. Surf. Finish. Soc. Jpn..

[36] Okamura Y. (2007). Environmental load reducing technologies in electroless plating process. J. Surf. Finish. Soc. Jpn..

[37] Watanabe K. (1999). Chromium plating. Hyomen Gijutsu.

[38] Shimpo R. (2012). Environmental corresponding of engineering hard plating. J. Jpn. Soc. Precis. Eng..

[39] Shimpo R. (2014). Properties of hard chrome plating. J. Surf. Finish. Soc. Jpn..

[40] Onodera H. (2018). Current status of trivalent chromium plating and its overseas trends. J. Surf. Finish. Soc. Jpn..

[B41-polymers-16-00224] 41.By courtesy of Nippon Paper Industries, Chiyoda-ku, Japan, 16 May 2023.

[42] (2012). Geometrical Product Specifications (GPS)—Surface Texture: Areal—Part 2: Terms, Definitions and Surface Texture Parameters.

[43] Rasband W.S. (1997–2020). ImageJ.

[44] Haeri M., Haeri M. (2015). ImageJ plugin for analysis of porous scaffolds used in tissue engineering. J. Open Res. Softw..

[45] Tosaka M. (2021). Basic knowledge of wide-angle X-ray diffraction. Nippon Gomu Kyokaishi.

[46] Doi T. (2015). Acting mechanisms of citric acid on the properties of nickel citrate electroplated film. Bull. Tokyo Metrop. Ind. Technol. Res. Inst..

[47] Takeichi Y. (2014). Tribology of polymer materials. J. Surf. Finish. Soc. Jpn..

[48] Isogai A., Onabe F. (1997). Cellulose: It’s higher-order structures and functionalities. Sen’i Kikai Gakkaishi (J. Text. Mach. Soc. Jpn.).

[49] AI-Jawhari I.F.H., Mallakpour S., Hussain C.M. (2021). Polymer Nanocomposite Matrix-Based Nanoproducts. Handbook of Consumer Nanoproducts.

[50] Isogai A. (2009). TEMPO-oxidized cellulose nanofibers. Kobunshi.

[51] Hase A. (2014). Research cases and trends of wear mechanism. J. Surf. Finish. Soc. Jpn..

[52] Kikuchi K., Kamiya O., Saito Y., Kumagai K. (1998). Running-in behavior of repeated dry wear on metals. J. Soc. Mater. Eng. Resour. Jpn..

[53] Mizumoto M., Usami K. (1994). [Why wear occuers] Mamou ha naze okoru noka (in Japanese). Turbomachinery.

[54] Tsujioka M. (2012). The technological trend and application of environment-friendly hard coating. J. Surf. Finish. Soc. Jpn..

[55] Mori H. (2012). Latest environmental technologies in the automobile industry (2). J. Surf. Finish. Soc. Jpn..

[56] Okano T. (2019). Technical trends of lubricants for cold forging. Plastos (Bull. JSTP).

